# Circumcision for prevention against HIV: marked seasonal variation in demand and potential public sector readiness in Soweto, South Africa

**DOI:** 10.1186/1748-5908-2-2

**Published:** 2007-01-25

**Authors:** Guy de Bruyn, Martin D Smith, Glenda E Gray, James A McIntyre, Russell Wesson, Gary Dos Passos, Neil A Martinson

**Affiliations:** 1 Perinatal HIV Research Unit, University of the Witwatersrand, Johannesburg, South Africa; 2 Department of Surgery, Chris Hani Baragwanath Hospital, and University of the Witwatersrand, Johannesburg, South Africa; 3 School of Medicine, Johns Hopkins University, Baltimore, MD, USA

## Abstract

The public sector delivery of male circumcision in the only public sector hospital in Soweto, South Africa was examined to gauge local capacity to deliver this procedure as an intervention for prevention of HIV acquisition. During the period from July 1998 to March 2006, approximately 360 procedures were performed per annum. Striking seasonal variations and the relatively few procedures performed may create challenges for program planning, if male circumcision is increased to a level required to have an impact on the incidence of HIV among this population.

## Findings

A recently completed randomized controlled trial of male circumcision (MC) for the prevention of HIV acquisition demonstrated that MC reduces the risk of HIV infection [1], confirming similar findings from many prior observational studies [2]. At the efficacy and cost of the procedure reported in the trial, MC may be cost-saving as a public health intervention [3]. These findings add to the options for personal prevention of HIV acquisition, and support the addition of MC as a component of prevention programs in countries with a high prevalence of HIV. In the absence of UNAIDS endorsement to back policy and program development [4], implementation issues need to be debated.

However, to have an impact, a large proportion of the male population would have to be circumcised. Indeed, this conclusion is supported by epidemic modeling for the population of Soweto [5], and at a country level in South Africa [6]. The current live male birth cohort in Soweto numbers approximately 15,000 per year (J. McIntyre, personal communication). The prevalence of MC is estimated to be between 20% and 35%, based on surveys in communities within 100 km of Soweto [7,8]. Population coverage of 60% of males within a birth cohort, without expanding to other uncircumcised men, would require at least 8,000 procedures per year. Additionally, MC appears to be acceptable to the majority of uncircumcised men in this area, if found to be beneficial in the prevention of HIV/STI's [7,8]. Partner preferences are commonly cited as a reason for adult men undergoing the procedure, because women find it acceptable as well.

Furthermore, South African investigators will soon initiate clinical research studies in HIV prevention, such as large HIV vaccine efficacy studies involving several thousand male participants. One of these trial sites is in Soweto. The ethical justification for the adoption of such measures as part of the standard of prevention for trial participants is still being debated, but clinical trial investigators would likely facilitate referral to medical services rather than traditional services, potentially adding a further burden to existing circumcision facilities [9,10].

However, it is currently unclear what capacity exists to supply circumcision on a scale to have an impact on HIV transmission in Soweto, a large urban township in the Johannesburg metropolitan area of South Africa with a population of approximately 1.1 million. To assess their existing medical capacity to undertake a large-scale circumcision program, we audited the procedure logs of the operating rooms of a 3,000-bed, public-sector hospital in Soweto, the only one serving this community, to document how many circumcision procedures were provided in recent years.

The numbers of circumcision procedures were retrieved from operating room logs for those procedures performed at Chris Hani Baragwanath Hospital by adult general surgical services between July 1998 and March 2006, and by the pediatric surgery service between July 2003 and March 2006. The indication for circumcision was not available from the logs.

**Figure 1:**
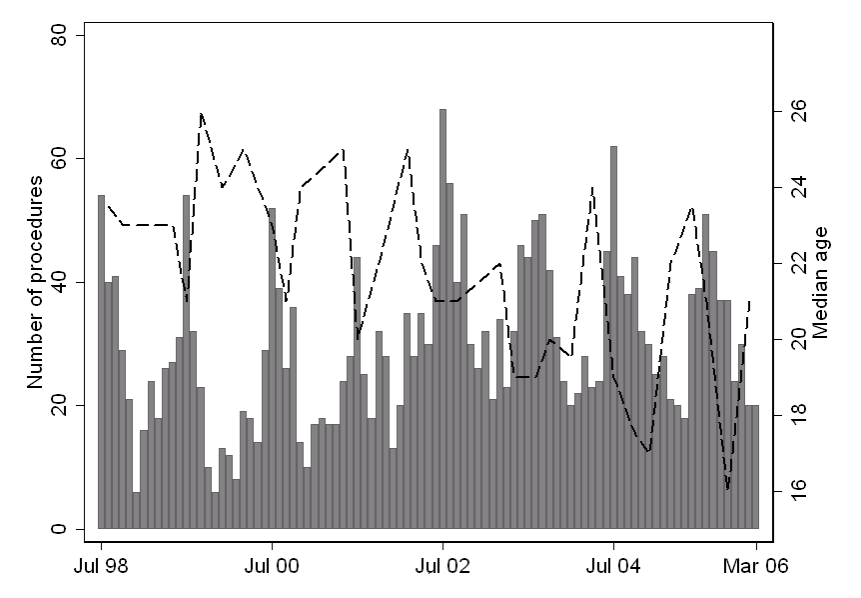
**Circumcision procedures performed by general surgical services, Chris Hani Baragwanath Hospital, Soweto, Jul 1998 – Mar 2006**. Bars represent number of procedures performed per month. Line represents median age (in years) of patients by month.

The survey found that 2,786 procedures were performed in the 93 continuous months of adult general surgical procedure logs available for review (mean ± standard deviation, 30 ± 13.1/month). The mean age of patients was 22.6 years (SD 8.8). The number of procedures varied strongly by season (Edwards test, *p *< 0.0001), being greatest in the winter months, with marked peaks in July or August and low rates in the summer months of December and January. The median age of males undergoing procedures also varied by season, with the median age being lower during peak months compared to months with lower rates (Figure 1). The median age showed a significant decline over the period (Cuzick test, *p *< 0.001).

The survey also found that 335 procedures were performed over the 33 continuous months of pediatric surgery procedure logs reviewed (mean, 10.2/month). One hundred and fifty-five (46.3%) procedures were performed on children between the age of one and five years. By contrast to the adult patients, no seasonality was noted for pediatric circumcisions (Edwards test, *p *= 0.355).

Public sector capability in Soweto exists to perform circumcisions on both boys and men. The numbers of MCs performed on men reflect the interaction between demand and supply for elective procedures, irrespective of a clinical indication, while surgery among pediatric patients would have been performed to treat a clinical indication. As indicated by current utilization, capacity would have to be substantially improved to deal with the additional caseload, estimated to be in the thousands per annum, if circumcision is to have a public health impact. Certainly, just the additional numbers that may be referred by large HIV prevention clinical trials would be equal to the total annual caseload. The striking variation in the number of procedures performed per month poses challenges to planning services and responding to demand. We have no current explanation for the observed seasonality of elective MC. Possible explanations include increased scheduling during school vacations. However, the numbers of procedures in July, for instance, do not vary according to public school holidays. We speculate that these patterns correspond to the timing of circumcision, as practiced in traditional or cultural rites of passage [11,12].

Alternatives to the provision of these services by specialist and trainee surgeons, such as local general practitioners or nurse practitioners, should be urgently explored if appropriate population coverage of circumcision is to be achieved. Current data on the number of procedures performed in Soweto in a traditional setting are not available, and these data will be an important component for developing programs and plans for innovative delivery solutions. Moreover, the concerns raised about potential diminished acceptability of MC if procedures are performed by female providers also would need to be further examined [13].

## Competing interests

The author(s) declare that they have no competing interests.

## Authors' contributions

Conception of study: GdB, MDS, NAM; Study Design: GdB, MDS, NAM; Acquisition of data: RW, GdP; Analysis and interpretation of data: GdB, MDS, GG, JAM, NAM; Drafting of manuscript: GdB, NAM; and Critical revision of manuscript: GdB, MDS, RW, GdP, NAM. All authors approved the final submitted version.
